# Early Starvation Contributes to the Adaptive Capacity of *Corythucha marmorata* (Uhler), an Emerging Pest in China

**DOI:** 10.3390/biology11010080

**Published:** 2022-01-05

**Authors:** Wei Zhou, Wenlong Chen

**Affiliations:** Guizhou Provincial Key Laboratory for Agricultural Pest Management of the Mountainous Region, Institute of Entomology, Scientific Observing and Experimental Station of Crop Pest in Guiyang, Ministry of Agricultural and Rural Affairs, Guizhou University, Guiyang 550025, China; Zw18786131893@126.com

**Keywords:** food shortage, development, survival, supercooling capacity, *Corythucha marmorata*

## Abstract

**Simple Summary:**

According to renowned Chinese medical saint Quan Wan, of the Ming Dynasty, “If you want to keep your child healthy, make him stay hungry occasionally”. In addition to lamenting the ancients’ theory of health preservation, we also frame this question in the context of insects of more distant origins. How did lace bugs adapt to starvation and low temperatures when they spread around the world? What influence do these abiotic stresses have? Driven by our desire to answer these questions, we designed a study in which nymphs newly born on different hosts were subjected to starvation treatment for differing periods. Origin food was then continuously provided until the end of their life. The total development time, survival, number of eggs, and supercooling capacity were recorded. Overall, our results provide an increased understanding of long-term post-starvation responses of insects to food limitations, particularly in determining survival.

**Abstract:**

Food shortages severely reduce the prospects of insect survival in natural settings, including in the case of herbivorous insects. However, the early starvation experience of some insects has positive effects throughout their entire lifespan. It is important to discuss the effects of refeeding and host plants on the capacity of herbivorous insects to adapt to starvation and low temperatures, considering that starvation resistance is expected to show some degree of adaptive phenotypic plasticity. We tested the relationship between host plant, starvation, and the supercooling capacity of the invasive pest *Corythucha marmorata*. In particular, we highlighted how early starvation affects the refeeding and recovery phases. Among the various range of hosts, the chrysanthemum lace bug has the fastest growth rate on *Helianthus annuus*, and the strongest supercooling capacity on *Symphyotrichum novi-belgii*. Especially, starvation for 2 days increases the rates of survival, development, and number of eggs upon refeeding, in comparison to no starvation. A 3-day starvation period in the nymphal stage significantly increased the supercooling capacity of 5th instar nymphs and adults, as observed in our study.

## 1. Introduction

Early-life starvation has been documented to have both positive and negative effects as determined by the species and environment [1,2,3]. The majority of studies support the influence of the latter, which includes the effects on insect survival rate [4], maximum body size [5], and reproductive output [6]. Meanwhile, a curious phenomenon is observed in many insects, which seems to improve the supercooling capacity of insects fed different host plants on account of changes in fitness [5,7,8,9]. Specifically, they increase their supercooling capacity by lowering their supercooling point (SCP) and freezing point (FP), in order to survive [10,11]. Generally, lower SCP and FP represent the greater cold tolerance to survive in an ultra-low temperature environment [11]. As is known, colonies in the juvenile stage are more likely to suffer from food deprivation because of their poor mobility [12,13]. Importantly, considering the seasonal changes associated with cold temperatures, what are the persistent impacts of starvation on the rest of the insect’s life? Some studies provide compelling evidence that starvation has a prolonged influence that can extend to subsequent developmental stages [14,15,16]. Similarly, we predicted that insects subjected to starvation in the immature stage would have superior growth performance as they develop because of their prior starvation experience. The exact benefits likely vary according to the different host plant species. However, no previous studies have investigated starvation and cold resistance in connection with host variety.

The chrysanthemum lace bug, *Corythucha marmorata* (Uhler), is reported as a notorious agricultural pest worldwide [17,18] and attacks chrysanthemums, major landscape ornamentals that play a significant role in greenhouses and the nursery industry [19,20]. Other hosts include sweet potatoes and Jerusalem artichokes [21,22,23,24,25,26], both of which have a high economic value as they are utilized in many fields, such as in the food and chemical industries and in medical treatment [27]. Lace bug nymphs and adults feed on the underside of leaves, removing cell contents from the upper palisade parenchyma layer and causing chlorosis and stippling. The sprouting leaves can appear bronzed or bleached in heavy infestations, and early leaf abscission can occur [28]. In some occurrences, especially in cultivated *Compositae* species, the entire plant turns yellow and dies in segments after becoming infested, which seriously affects the growth of plants and the appearance of the landscapes containing these plants [26].

The lace bug is an insect herbivore belonging to the group of polyphagous pests [18,29]. Its host range includes a wide range of species over five plant genera, though species of other genera have also been reported as hosts, and species reported as hosts include, but are not limited to, *Ipomoea batatas*, *Ambrosia trifida*, *Artemisia princeps*, *Bidens pilosa*, *Conyza canadensis*, *Gazania rigens*, *Helianthus annuus*, *Helianthus tuberosus*, *Solidago altissima*, and *Symphyotrichum novi-belgii* [21,22,29,30,31]. Concomitant feeding is mostly dependent on *S. altissima* in the native ranges of North America [32], and on *A. trifida* on Shikoku Island, Japan [21]. Yet, this dangerous pest is also considered a successful invader and prefers to feed on *Compositae* cash crops, such as *H. annuus, H. tuberosus*, and *S. novi-belgii* in China [22,26,33]. *C. marmorata* has indeed caused great damage in eight Chinese provinces, up to the year 2020 (i.e., Shanghai [22], Hubei [24], Zhejiang [34,35], Taiwan [35], Jiangxi [36], Jiangsu [37], Henan [38], and Guizhou [33]). Since it was first discovered in Shanghai in 2010, this pest has attacked commercial *Compositae* for 10 consecutive years. It is clear that, in Japan, the rapid speed of diffusion has surpassed that of performance improvements [21].

During diffusion, the lace bug undoubtedly encounters food shortages as weak fliers [18]. Overwintering adults emerge in early spring and oviposit on the first available basal rosettes. Eggs are deeply embedded in the lower leaf surface along the midrib. In Maryland, up to five generations per year are common [18]. In China, in the city of Wuhan, there are five generations of lace bug per year [37]. They overwinter as adults on dead leaves, weeds, or rhizosphere topsoil by the end of March and early April in the following year, and there is another period of overwintering for adults beginning at the end of October. Overlapping generations can be found in all seasons of the year. The most serious damage occurs from July to September, when adults begin to mate and lay eggs 7 days after eclosion. Most of the eggs are scattered in the tissues along the main vein on the back of the host leaf. Nymphs occasionally move between leaves to prevent food shortages. The tremendous starvation resistance of this intruder provides an ideal model for our study on the responses and effects of starvation.

In the present experiment, laboratory analysis was conducted to assess the survival rate, development time, reproduction, and supercooling capacity of *C. marmorata*, whose 1st instar nymphs were subjected to starvation for different days and commenced refeeding, separately, on four different types of host plants. We examined the impact of early hunger on subsequent life activities, thus filling in the apparent plasticity index of the insect refeeding stage, and providing a reference for assessing the chrysanthemum lace bug when it causes damage by transferring to a greater number of host plants.

Herein, we answer the following questions: (1) what are the persistent impacts of starvation during the whole life of *C. marmorata*? (2) Are there differences between having annual hosts or perennial hosts for lace bugs in adapting to starvation stress?

## 2. Materials and Methods

### 2.1. Experimental Insects and Host Plants

*C*. *marmorata* adults were collected in 2018 from *H. annuus*, *H. tuberosus*, *S. novi-belgii*, and *A. trifida* plants located in Guiyang, Guizhou, China. Adult colonies were maintained at the Institute of Entomology, Guizhou University, Guiyang city, China, under the following conditions: 26 ± 1 °C and 65% relative humidity, and 14 h light/10 h dark conditions in an artificial chamber (Ningbo Jiangnan, Ningbo, China). In June 2019, newly emerged 1st instar nymphs (<2 h old) were collected from harvested eggs and raised in an artificial chamber to obtain the F_2_ generation. The 1st instar nymphs (<2 h old) from the F_1_ generation were reared in Petri dishes (9 cm) on the fresh leaves of four plants in artificial conditions, as mentioned above. Old leaves were replaced with fresh leaves and filter paper was replaced where necessary, in order to ensure sufficient food availability and prevent desiccation. Emerged adults of *C*. *marmorata* were collected and placed into clean Petri dishes. Adults were raised on fresh leaves and at controlled conditions, as mentioned above, to obtain the F_2_ generation. First instar nymphs from the F_2_ generation were divided into different groups to determine the starvation and supercooling capacity effects.

Due to the lace bug being multivoltine, the population density of the bugs on annual host plants usually increases with each successive advancing generation, and adults migrate to nearby host plants. Then, the adult bug overwinters under the leaves of perennial host plants. We selected three species as annual hosts and one species as a perennial host, to simulate the environment of host transformation. Host treatments were selected from a list of documented Chinese hosts for *C. marmorata*, with priority given to those species and varieties that occur in subtropical (i.e., southwest China) climates. Through personal field observation, the four most seriously harmful plants by the lace bug in Guizhou Province were identified. We planted *H. annuus* and *A. trifida* in the field. The experimental plant leaves of *H. tuberosus* and *S. novi-belgii* were collected from our campus. A large collection of all host plants had been maintained during rearing in the school and without applying pesticides.

### 2.2. Observation of Development on Four Host Plants of C. marmorata

First instar nymphs from the F_2_ generation were divided into four groups according to the difference of hosts. Ninety individuals were placed in each group. A total of 360 individuals participated in this experiment. Each group of bugs was fed on one species of host plant in order to obtain the F_1_ to F_2_ generations, and to measure the development time, survival rate, sex ratio, and number of eggs. In the F_2_ generation, we checked the number of dead lace bugs per day with a fine brush. The development time was defined as days from the 1st instar to adult emergence. Thus, we confirmed the sex of emerged adults (<24 h old) and made them mate (female/male = 1:1). If the males die, replenish the newly emerged males in time to ensure that they can lay eggs. The number of eggs was recorded daily, from the beginning of oviposition to the death of females to calculate fecundity of per female during lifetime.

### 2.3. Measurement of Early Starvation on Development of C. marmorata

First instar nymphs from the F_2_ generation were collected for five experiments (development time, survival rate, sex ratio, the number of eggs, and SCP/FP). Each experiment comprised four groups. Three groups were used as treated groups (without water and food). Thirty individuals were placed in each group. Three replicates were used for each treatment. Treated groups were marked as 1 day’s starvation (1DS), 2 days’ starvation (2DS), and 3 days’ starvation (3DS). However, the control group was given fresh host leaves and clean fresh water. In order to measure the early starvation effect on later life stages, we conducted the same observation, as shown in Section 2.2, when restoring supply hosts separately for starved lace bugs. The sample size for the sex ratio were as follows: 328 adults on control, 319 adults on 1DS, 343 adults on 2DS, and 313 adults on 3DS. For the number of eggs laid, we recorded 360 females on control and 120 females on each treated group.

### 2.4. Determination of the Supercooling and Freezing Point

Fifth instar nymphs (<2 h old), male and female adults (<24 h old) that had emerged from starvation-treated groups (F_2_ generation), were selected for the determination of supercooling point (SCP) and freezing point (FP). Forty *C. marmorata* individuals were selected from *H. annuus*, *H. tuberosus*, *S. novi-belgii*, and *A. trifida*. The measurement was performed on isolated lace bugs placed inside a 1 mL Eppendorf PCR tube fixed to the tip of a thermocouple with scotch tape. The tube was sealed with plastic parafilm and cotton. The thermocouple was connected to a computer with built-in testing software for recording the temperature every second (SN: 2020007559, SUN-V, Beijing, China). The tube was placed inside an ultra-low temperature refrigerator of −40 °C (SN: 2020007560, DW-40L525, Qingdao, China). The SCP and FP values were displayed in real time on the software screen.

### 2.5. Data Analysis

All statistical analyses were conducted in IBM SPSS Statistics Version 21 (SPSS, Chicago, IL, USA) and graphs prepared in Prism 8.2.1 (GraphPad Software, Inc., San Diego, CA, USA). For all ANOVA analyses, residual plots were checked for normality (Shapiro–Wilk test) or homoscedasticity (Levene’s test) of the residuals. If the difference was significant, we used post hoc multiple comparisons of mean ranks for all groups (Duncan’s test).

## 3. Results

### 3.1. Shortened Development Time of C. marmorata Suffering from Early Starvation

**Figure 1:**
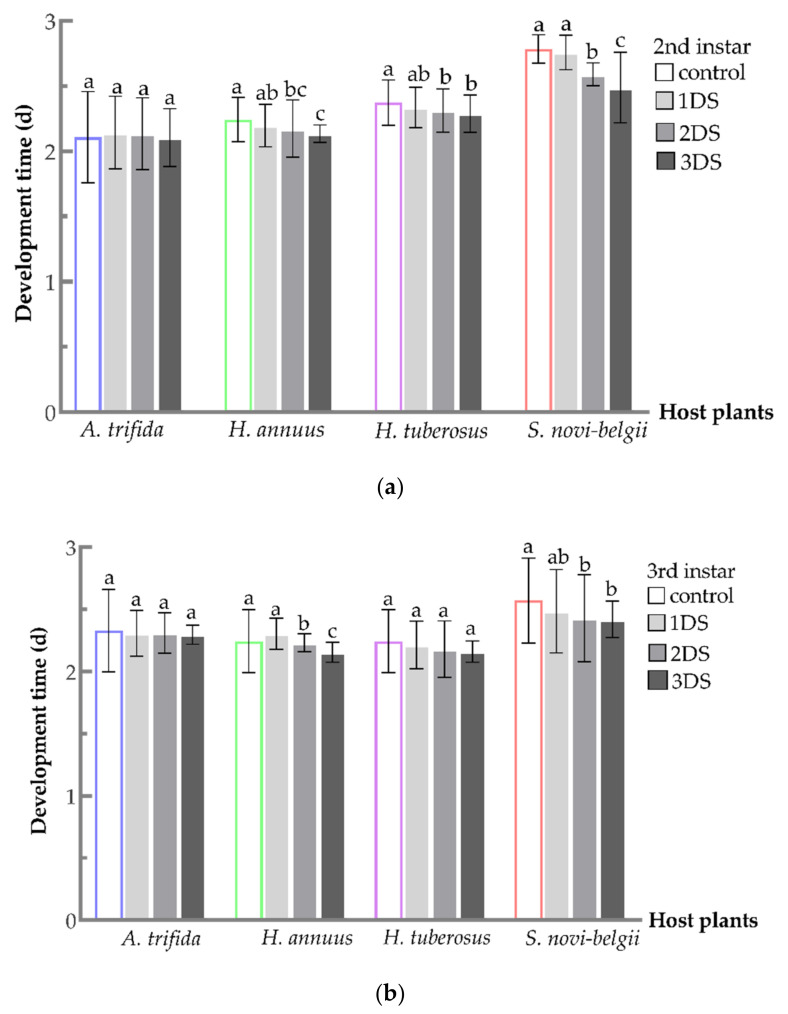

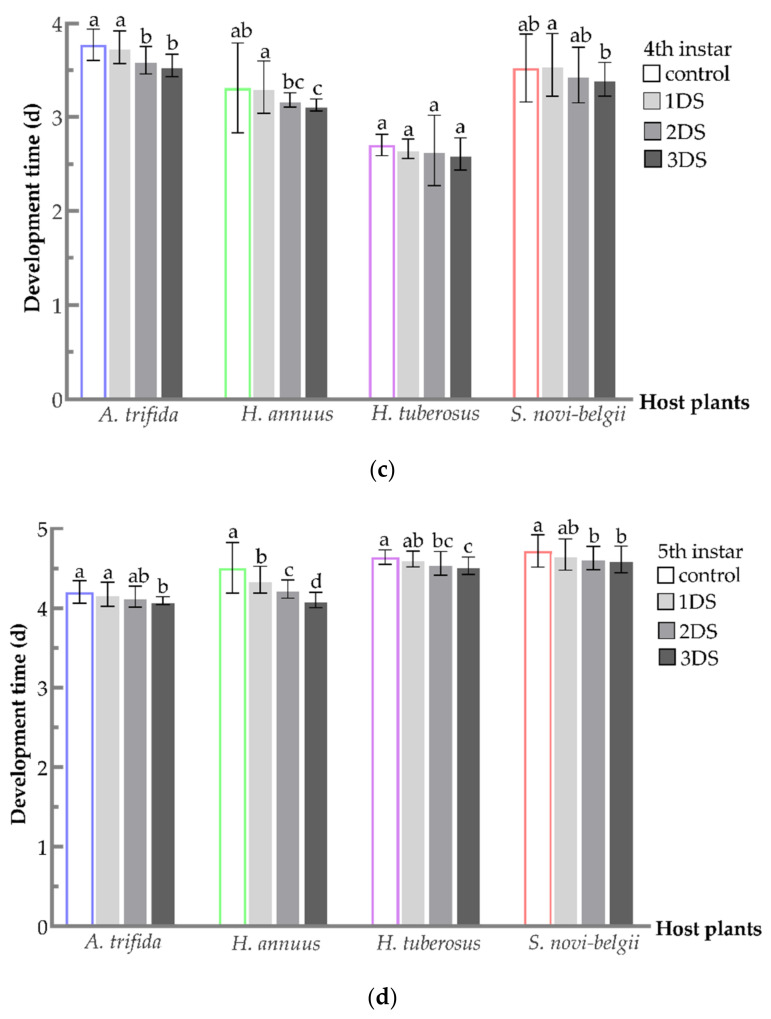
Development time of *C. marmorata* nymphs suffering from food deprivation on four host plants. (**a**) 2nd instar nymph; (**b**) 3rd instar nymph; (**c**) 4th instar nymph; and (**d**) 5th instar nymph. Control: the group offered host leaflets continuously without food deprivation; 1DS: group subjected to 1 day’s starvation; 2DS: group subjected to 2 days’ starvation; and 3DS: group subjected to 3 days’ starvation. Values are the mean ± SD. Different lowercase letters “a, ab, b, bc, c, d” in one species of host plants indicate significant differences (one-way analysis of variance followed by Duncan’s test, *p* < 0.05).

The development time of *C. marmorata* nymphs on four host plants is presented in Figure 1. As expected, differences were observed after being subjected to starvation. The development time of the 2nd instar nymphs fed on *H. annuus* (F_3,292_ = 4.3559, *p* = 0.0051), *H. tuberosus* (F_3,292_ = 3.2972, *p* = 0.0209), and *S. novi-belgii* (F_3,296_ = 58.1881, *p* < 0.001), subjected to 2 and 3 days of food deprivation, was significantly lower when compared to 1 day starvation and the control treatment (Figure 1a). However, the 2nd instar nymphs subject to 1, 2, and 3 days of food deprivation fed on *A. trifida* were not significantly different from each other and with control treatment (F_3,292_ = 0.3236, *p* = 0.8083). In the case of 2 and 3 days of food deprivation, the development time of the 3rd instar nymphs fed on *H. annuus* (F_3,259_ = 7.3216, *p* = 0.0001) and *S. novi-belgii* (F_3,266_ = 3.0523, *p* = 0.0290) was significantly less when compared to the first day and control treatments (Figure 1b). By contrast, the 3rd instar nymphs fed on *A. trifida* (F_3,252_ = 0.1726, *p* = 0.9148) and *H. tuberosus* (F_3,254_ = 1.6489, *p* = 0.1786), subjected to 2 and 3 days of food deprivation, were not significantly different when compared to day 1 and control treatment. Furthermore, the 4th instar nymph fed on *H. annuus* (F_3,229_ = 4.3548, *p* = 0.0053), *S. novi-belgii* (F_3,248_ = 2.2605, *p* = 0.0280), and *A. trifida* (F_3,231_ = 19.6319, *p* < 0.001) after 3 days of food deprivation, had a significantly shortened development time when compared to 1 and 2 days of starvation and control treatment (Figure 1c). However, no significant difference in development time was observed in 1, 2, and 3 days and control on *H. tuberosus* (F_3,232_ = 1.1287, *p* = 0.3382) (Figure 1c). For the 5th instar nymph fed on *H. annuus*, the development time was significantly shortened with 1, 2, and 3 days of food deprivation when compared to the 1st day and control treatment (F_3,209_ = 24.3980, *p* < 0.001) (Figure 1d). Moreover, a significant difference was observed in the development time on nymphs fed on *H. tuberosus* (F_3,218_ = 7.1214, *p* = 0.0001), *S. novi-belgii* (F_3,238_ = 3.6379, *p* = 0.0135), subjected to 2 and 3 days of food deprivation. However, the development time of nymphs fed on *A. trifida* was significantly shortened after 3 days of food deprivation (F_3,215_ = 3.2945, *p* = 0.0214).

**Figure 2:**
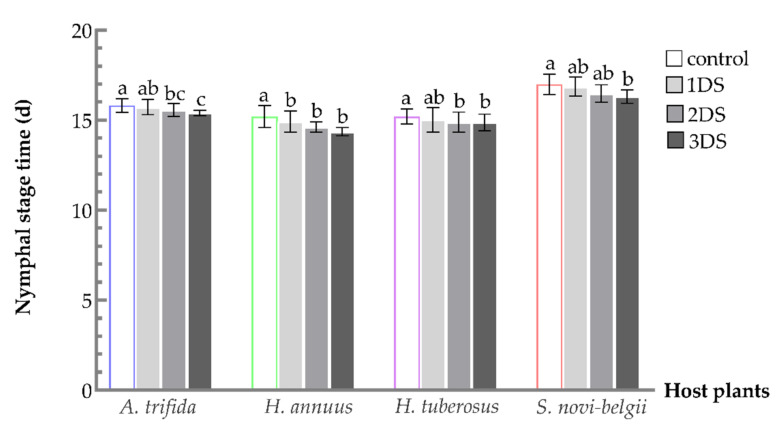
Total nymphal stage time of *C. marmorata* suffering from food deprivation on four host plants. Control: the group offered host leaflets continuously without food deprivation; 1DS: the group subjected to 1 day’s starvation; 2DS: the group subjected to 2 days’ starvation; and 3DS: the group subjected to 3 days’ starvation. Values are the mean ± SD. Different lowercase letters “a, ab, b, bc, c” in one species of host plants indicate significant differences (one-way analysis of variance followed by Duncan’s test, *p* < 0.05).

For total nymphal stage time on four hosts, a significant decrease was observed in 2 and 3 days of food deprivation, compared to the control (*S. novi-belgii*, F_3,238_ = 17.8624, *p* < 0.001; *A. trifida*, F_3,215_ = 7.4243, *p* = 0.0001; *H. tuberosus*, F_3,219_ = 3.9700, *p* = 0.0088; and *H. annuus*, F_3,210_ = 19.1379, *p* < 0.001) (Figure 2). Moreover, with the prolongation of food deprivation time, nymphs spent a shorter time developing into adults. After suffering from food deprivation, nymphs fed on *S. novi-belgii*, *A. trifida*, *H. tuberosus*, and *H. annuus* saved 0.68 d, 0.42 d, 0.33 d, and 0.84 d, respectively.

### 3.2. Sustained High Survival Rate of C. marmorata Suffering from Early Starvation

**Figure 3:**
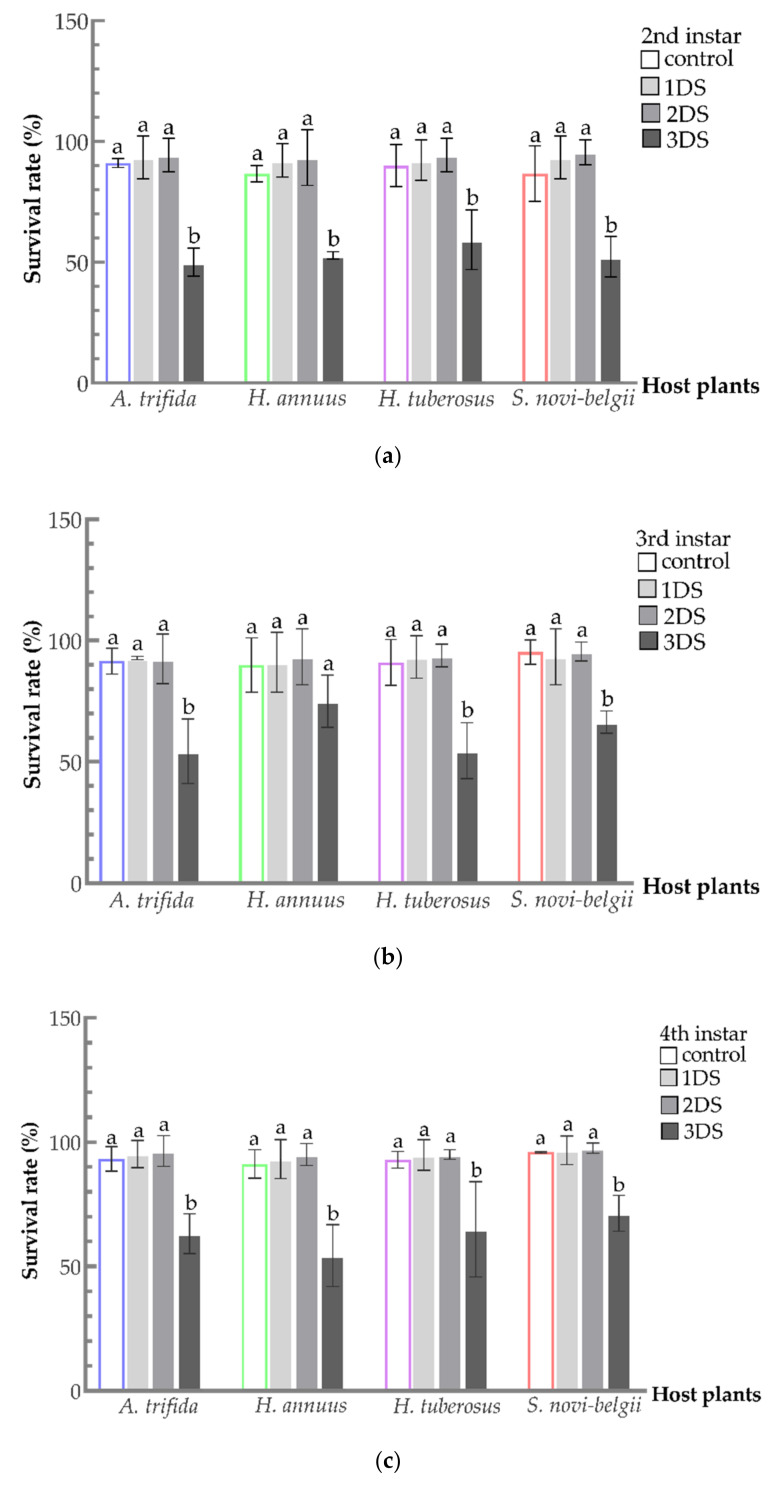

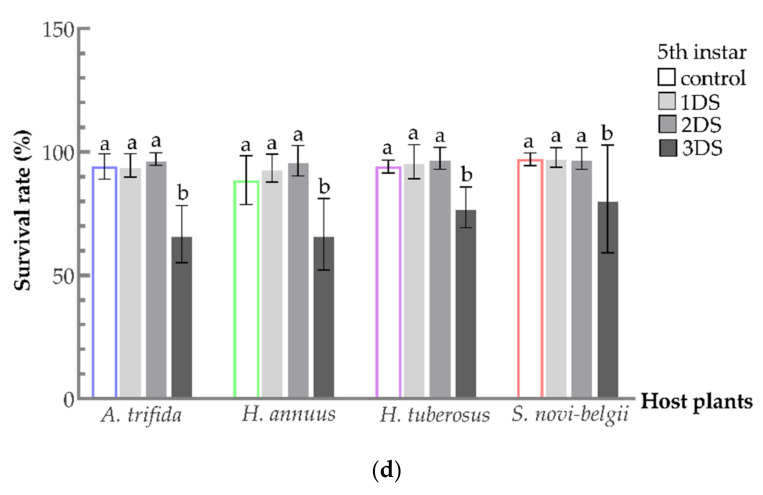
Survival rate of *C. marmorata* nymph suffering from food deprivation on four host plants: (**a**) 2nd instar nymph; (**b**) 3rd instar nymph; (**c**) 4th instar nymph; and (**d**) 5th instar nymph. Control: the group offered host leaflets continuously without food deprivation; 1DS: the group subjected to 1 day’s starvation; 2DS: the group subjected to 2 days’ starvation; and 3DS: the group subjected to 3 days’ starvation. Values are the mean ± SD. Different lowercase letters “a, b” in one species of host plants indicate significant differences (one-way analysis of variance followed by Duncan’s test, *p* < 0.05).

Most nymphs can survive after early food deprivation (50–80.95%) (Figure 3). A similar increase in the survival rate of 2nd–5th instar nymphs was observed after 1 and 2 days of food deprivation; however, the difference was not significant. By contrast, a significant decrease in the survival rate was observed after 3 days of food deprivation (second instar nymphs: *A. trifida*, F_3,8_ = 34.1235, *p* = 0.0001; *H. annuus*, F_3,8_ = 22.6897, *p* = 0.0003; *H. tuberosus*, F_3,8_ = 9.4270, *p* = 0.0053; and *S. novi-belgii*, F_3,8_ = 15.8818, *p* = 0.0010. Third instar nymphs: *Ambrosia trifida*, F_3,8_ = 13.8993, *p* = 0.0015; *H. annuus*, F_3,8_ = 1.5737, *p* = 0.2700; *H. tuberosus*, F_3,8_ = 13.6130, *p* = 0.0017; and *S. novi-belgii*, F_3,8_ = 12.4275, *p* = 0.0022. Fourth instar nymphs: *A. trifida*, F_3,8_ = 19.6087, *p* = 0.0005; *H. annuus*, F_3,8_ = 16.9087, *p* = 0.0008; *H. tuberosus*, F_3,8_ = 6.1455, *p* = 0.0180; and *S. novi-belgii*, F_3,8_ = 21.6572, *p* = 0.0003. Fifth instar nymphs: *A. trifida*, F_3,8_ = 13.0263, *p* = 0.0019; *H. annuus*, F_3,8_ = 5.7681, *p* = 0.0212; *H. tuberosus*, F_3,8_ = 7.1879, *p* = 0.0117; and *S. novi-belgii*, F_3,8_ = 1.5657, *p* = 0.2718). Second instar nymphs fed on *A. trifida* had the highest survival rate among hosts in the control (91.11%); however, this position was replaced after 2 days of deprivation on *S. novi-belgii* (95.56%) (Figure 3a). Interestingly, compared with other hosts, 3rd (except for 3 days of food deprivation), 4th, and 5th instar nymphs fed on *Symphyotrichum novi-belgii* all had the highest survival rate in the control and food deprivation groups (Figure 3b–d). Additionally, the 5th instar nymphs had the highest survival rate on *S. novi-belgii* (97.03%) in the control (Figure 3d). After 2 days of food deprivation, this advantage was maintained although not significant (97.44%). Then, the survival rate decreased to 80.95% after 3 days of food deprivation.

### 3.3. Similar Sex Ratio of C. marmorata Suffering from Early Starvation

**Figure 4:**
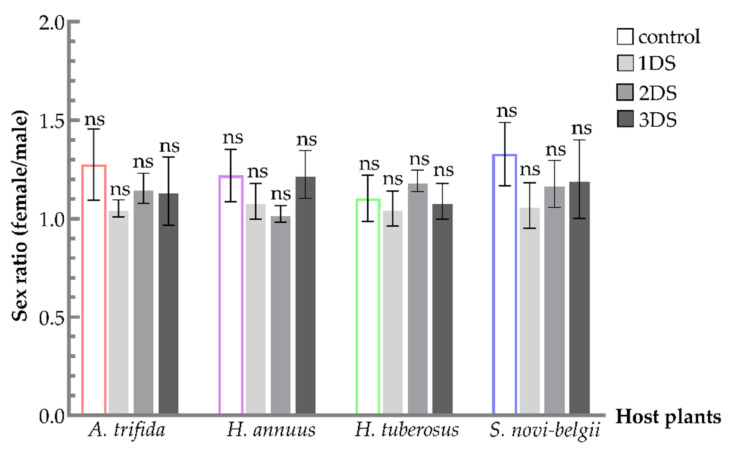
Sex ratio of *C. marmorata* suffering from food deprivation on four host plants. Control: the group offered host leaflets continuously without food deprivation; 1DS: the group subjected to 1 day’s starvation; 2DS: the group subjected to 2 days’ starvation; and 3DS: the group subjected to 3 days’ starvation. Values are the mean ± SD. Letters “ns” in one species of host plants indicate differences that are not significant (one-way analysis of variance followed by Duncan’s test, *p* > 0.05).

The sex ratio fluctuated and remained greater than 1 after food deprivation. After the early food deprivation of nymphs, the number of female emergences still outnumbered that of males (*S. novi-belgii*, F_3,8_ = 1.4714, *p* = 0.2938; *A. trifida*, F_3,8_ = 1.4273, *p* = 0.3048; *H. tuberosus*, F_3,8_ = 1.2895, *p* = 0.3426; and *H. annuus*, F_3,8_ = 2.7536, *p* = 0.1120) (Figure 4). The sex ratios for groups with early food deprivation showed different tendencies, according to feeding on the four different hosts. Adults fed on *S. novi-belgii* had higher sex ratios than that of *A. trifida*, in all experimental groups. By contrast, the sex ratio on *H. annuus* and *H. tuberosus* was lower than *A. trifida* in the control. After 1 and 3 days of food deprivation of nymphs, the sex ratio for *H. annuus* was higher than *A. trifida*. Similarly, the sex ratio for *H. tuberosus* was higher after 2 days of food deprivation.

### 3.4. Increased Number of Eggs Laid by Females Suffering from Early Starvation

**Figure 5:**
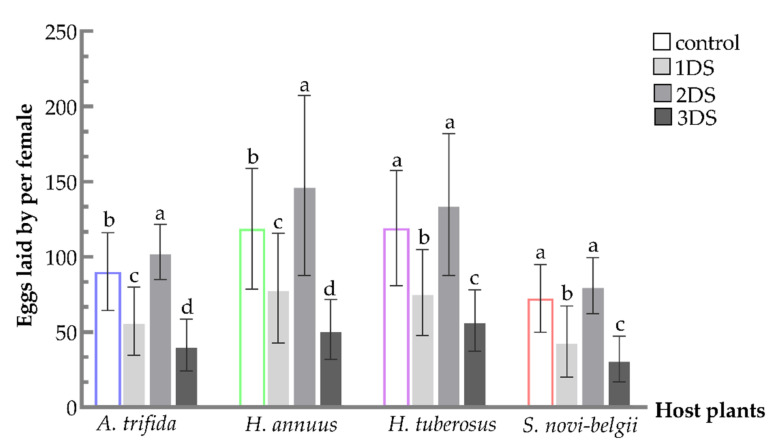
Eggs laid by per female of *C. marmorata* suffering from food deprivation on four host plants. Control: the group offered host leaflets continuously without food deprivation; 1DS: the group subjected to 1 day’s starvation; 2DS: the group subjected to 2 days’ starvation; and 3DS: the group subjected to 3 days’ starvation. Values are the mean ± SD. Different lowercase letters “a, b, c, d” in one species of host plants indicate significant differences (one-way analysis of variance followed by Duncan’s test, *p* < 0.05).

A significant increase was observed after 2 days of food deprivation on *A. trifida* and *H. annuus* (Figure 5). For females fed on *A. trifida*, the number of eggs increased compared to the control (103.3 ± 3.33). For females fed on *H. annuus*, the number of eggs increased compared to the control (147.5 ± 10.92). However, the increase was not significant on *Helianthus tuberosus* (134.87 ± 8.60) and *S. novi-belgii* (80.9 ± 3.40). Overall, females fed on *Helianthus annuus* and *H. tuberosus* spawned more eggs than that of *A. trifida*, and the number of eggs significantly decreased after 1 and 3 days of food deprivation compared with the control on 4 hosts (*A. trifida*, F_3,116_ = 54.6796, *p* < 0.001; *H. annuus*, F_3,116_ = 31.0582, *p* < 0.001; *H. tuberosus*, F_3,116_ = 31.5439, *p* < 0.001; and *S. novi-belgii*, F_3,116_ = 39.0093, *p* < 0.001).

### 3.5. Decreased SCP and FP of C. marmorata Suffering from Early Starvation

**Figure 6:**
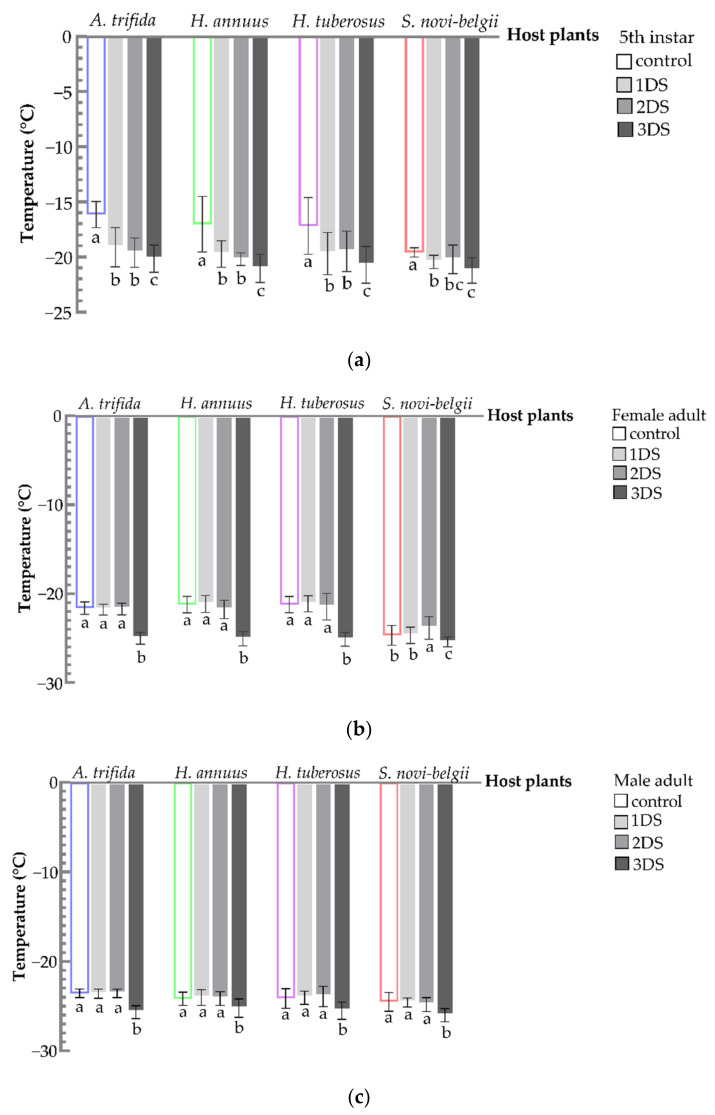
Supercooling point of *C. marmorata* suffering from food deprivation on four host plants. (**a**) SCP of 5th instar nymph; (**b**) SCP of female adult; and (**c**) SCP of male adult. Control: the group offered host leaflets continuously without food deprivation; 1DS: the group subjected to 1 day’s starvation; 2DS: the group subjected to 2 days’ starvation; and 3DS: the group subjected to 3 days’ starvation. Values are the mean ± SD. Different lowercase letters “a, b, bc, c” in one species of host plants indicate significant differences (one-way analysis of variance followed by Duncan’s test, *p* < 0.05).

For 5th instar nymphs, a significant decrease in SCP was observed after 1, 2, and 3 days of food deprivation, compared to the control (*A. trifida*, F_3,156_ = 64.8850, *p* < 0.001; *Helianthus annuus*, F_3,156_ = 49.0256, *p* < 0.001; *H. tuberosus*, F_3,156_ = 21.6266, *p* < 0.001; and *S. novi-belgii*, F_3,156_ = 20.5772, *p* < 0.001) (Figure 6a). The SCP of 5th instar nymphs on *S. novi-belgii* reached the lowest point (−21.22 °C). For female and male adults, a significant decrease was only observed after 3 days of food deprivation (female: *A. trifida*, F_3,156_ = 254.6556, *p* < 0.001; *H. annuus*, F_3,156_ = 15.4071, *p* < 0.001; *H. tuberosus*, F_3,156_ = 133.0178, *p* < 0.001; and *Symphyotrichum novi-belgii*, F_3,156_ = 16.2718, *p* < 0.001. Male: *A. trifida*, F_3,156_ = 142.2062, *p* < 0.001; *H. annuus*, F_3,156_ = 16.1991, *p* < 0.001; *H. tuberosus*, F_3,156_ = 22.3125, *p* < 0.001; and *S. novi-belgii*, F_3,156_ = 30.7865, *p* < 0.001). No significant influence was observed on SCP of females and males after 1 and 2 days of food deprivation, compared to the control. Additionally, the SCP of females and males fed on *S. novi-belgii* was the lowest in four hosts after 3 days of food deprivation (Figure 6b,c).

**Figure 7:**
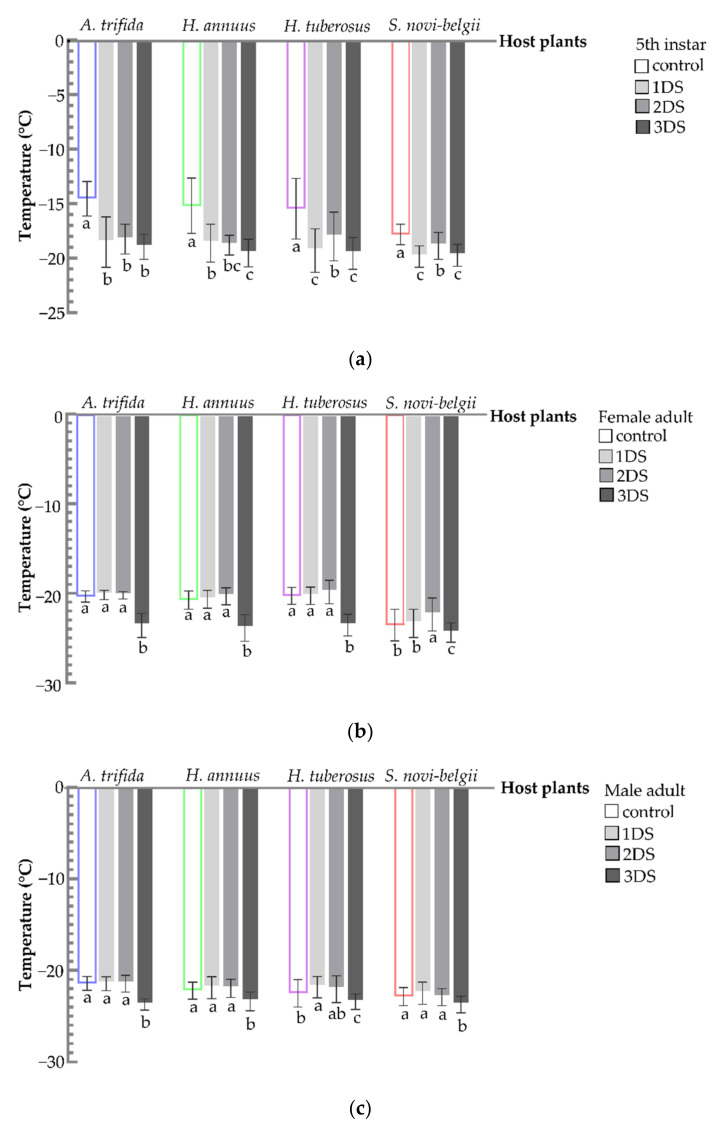
Freezing point of *C. marmorata* suffering from food deprivation on four host plants. (**a**) FP of 5th instar nymph; (**b**) FP of female adult; and (**c**) FP of male adult. Control: the group offered host leaflets continuously without food deprivation; 1DS: the group subjected to 1 day’s starvation; 2DS: the group subjected to 2 days’ starvation; and 3DS: the group subjected to 3 days’ starvation. Values are the mean ± SD. Different lowercase letters “a, ab, b, bc, c” in one species of host plants indicate significant differences (one-way analysis of variance followed by Duncan’s test, *p* < 0.05).

For 5th instar nymphs, a significant decrease in the FP was observed after 1, 2, and 3 days of food deprivation, compared to the control (*A. trifida*, F_3,156_ = 59.7271, *p* < 0.001; *H. annuus*, F_3,156_ = 50.8482, *p* < 0.001; *H. tuberosus*, F_3,156_ = 29.6885, *p* < 0.001; and *S. novi-belgii*, F_3,156_ = 32.0431, *p* < 0.001) (Figure 7a). For female and male adults, significantly decreased FP was also observed after 3 days of food deprivation compared to the control (female: *A. trifida*, F_3,156_ = 170.0031, *p* < 0.001; *H. annuus*, F_3,156_ = 85.0794, *p* < 0.001; *H. tuberosus*, F_3,156_ = 92.9745, *p* < 0.001; and *S. novi-belgii*, F_3,156_ = 10.8603, *p* < 0.001. Male: *A. trifida*, F_3,156_ = 86.4649, *p* < 0.001; *H. annuus*, F_3,156_ = 17.6631, *p* < 0.001; *H. tuberosus*, F_3,156_ = 12.2002, *p* < 0.001; and *Symphyotrichum novi-belgii*, F_3,156_ = 10.6032, *p* < 0.001) (Figure 7b,c). Thus, compared to the fed adults, the nymphs achieved more consistent values for SCP and FP across treatments after food deprivation.

## 4. Discussion

This research explored the effect of early food deprivation on subsequent development stages of *C. marmorata* and the relationship of stress to overall adaptability. Our results demonstrate that the nymphal stage development time, SCP, and FP decreased along with an increase in egg numbers for lace bugs after different food deprivations, compared to those that were fed. Nymphs suffering from 3 days of food deprivation showed a decreased survival rate, although most transitioned into adults. Due to early starvation, surviving 5th instar nymphs and adults showed lower SCP and FP on all four hosts, which is a common outcome. This indicates that early food deprivation did not limit the development and fecundity and, instead, partly contributed to the adaptive ability of this new invasive pest in China.

Food shortage is a ubiquitous abiotic stress that exerts a profound influence on the expansion of the insect population. Insects have the ability of exhibiting a plastic response to environmental stress. The theory of life history predicts that animals should increase their current reproductive efforts as the probability of survival decreases, i.e., up to the next reproductive opportunity. Recent studies concerning the biological characteristics of *C. marmorata* have provided data relevant for assessing its potential threat in China. Shen et al. [34] reported that the average nymphal stage time of *C. marmorata* was from 13.43 to 16.33 d on different host plants of *Solidago canadensis*, which was similar to our result of nymphal stage time on four host plants (from 14.36 to 16.31 d). Additionally, the stable survival rate after emerging into adults commonly supported this period, which is the critical stage for population growth of *C. marmorata*.

Our results suggest that starvation for a 2-day period, results in an increase in the development rate, survival rate, egg amount, and absolute value of SCP and FP. The results of a previous study support the notion of good adaptation in benign and stressful environments, due to alternative life-history patterns [18]. Moreover, *C. marmorata* nymphs grow a complex of strongly sclerotized with three-pronged spines to protect nymphal armature. To survive in food deprivation, *C. marmorata* females conceal their eggs in leaf tissue and spread their reproductive effort over time and area.

*C. marmorata* oviposition on *S. canadensis* was from 69.4 to 105 eggs per female; however, the average oviposition on *H. annuus* and *H. tuberosus* was 119 eggs per female. The possible reason may be that the leaves of *S. canadensis* are relatively small, and the space for spawning is also limited. The research of Zhu [26] showed cultivated hosts, such as chrysanthemums and sunflowers, suffered more severely. This was supported by our result that nymphs had a faster developmental rate and females laid more eggs on *H. annuus* and *H. tuberosus*. Otherwise, Yu et al. reported that the sex ratio of *Eocanthecona furcellata* (Wolff.) remained constant when rearing in different nymph densities, which is supported by our result that the sex ratio was not affected by food density and species [39,40,41].

Our study suggested that the development time and survival rate of the nymphs feeding on *S. novi-belgii* were significantly longer than those feeding on the other annual hosts, and 5th instar nymphs and adults had lower supercooling and freezing points. These results can be due to changes in water, glycogen, fat, and sorbitol contents in insect bodies after feeding on different plants. The lace bugs select annual hosts to feed on once they emerge as adults in the spring and summer. They grow quickly and lay a large number of eggs. As a result, the population is more likely to spread out and feed on many hosts in order to increase the invasion region. The difference is that they reside on perennial hosts throughout the winter and develop a significant supercooling capacity, resulting in a high survival rate and the population is more inclined to survive. This life history trade-off can be more visible under early hunger pressure. Overwintering adults had experience to adapt cold stress on perennial hosts than annual hosts. Our result of SCP and FP showed that *C. marmorata* have a great supercooling capacity to survive in ultra-low temperature environments (−26.01 °C), which was consistent with the prediction of Wang et al. [42] that the potential distribution was in 20° ~ 40° N. We also paid attention to the development and supercooling capacity performance (completed the whole life history by feeding four plants in one month). Obviously, *C. marmorata* had successfully invaded and would rapidly spread in China.

The effects of post-starvation refeeding on the development and supercooling capacity of *C. marmorata* subjected to low temperatures, were investigated in the present paper. The results showed that after starving the 1st instar nymphs, the starvation time had a significant effect on the survival, development, and SCP/FP of *C. marmorata*. This also prolonged the effect of the starvation and refeeding stage of *C. marmorata*. Yet, our investigation is an experimental study on the refeeding stage of *C. marmorata* at a constant temperature of 26 °C. A given package of life-history traits is the product of many interacting selection pressures that maximize individual fitness through evolutionary responses to trade-offs among traits. The cold tolerance (across different temperatures) of *C. marmorata* under low-temperature conditions should be further explored, in order to provide a basis for understanding its biology during winter. Collectively, our results suggest that starving *C. marmorata* for 2 days upon refeeding had a positive influence on its adaptive capacity.

## 5. Conclusions

Herein, we indicate that early starvation did not limit the development and fecundity and, instead, partly contributes to the adaptive ability of *C. marmorata* in China. Compared to the fed adults, nymphs achieved a more consistent supercooling capacity across treatments after being subjected to the same starvation period. Adults can have a better ability to recover after being stressed by starvation. These events include faster development and survival rates, higher fecundity, and deeper SCP and FP. We first reported the persistent impacts of starvation and refeeding in the nymphal stage of *C. marmorata*. Collectively, we report that starving *C. marmorata* for 2 days upon refeeding had a positive influence on its adaption capacity. These findings highlight the profound impact of refeeding after starvation, which can be helpful to comprehend the reasons for chrysanthemum lace bug large-scale invasion.

## Data Availability

The data presented in this study are available on request from the corresponding author.
